# Which Diet for Calcium Stone Patients: A Real-World Approach to Preventive Care

**DOI:** 10.3390/nu11051182

**Published:** 2019-05-27

**Authors:** Claudia D’Alessandro, Pietro Manuel Ferraro, Caterina Cianchi, Massimiliano Barsotti, Giovanni Gambaro, Adamasco Cupisti

**Affiliations:** 1 Department of Clinical and Experimental Medicine, University of Pisa, 56126 Pisa, Italy; dalessandroclaudia@gmail.com (C.D.); cate.cianchi@virgilio.it (C.C.); 2 U.O.C. Nefrologia, Fondazione Policlinico Universitario A. Gemelli IRCCS, Roma, Italia Università Cattolica del Sacro Cuore, 00168 Roma, Italy; pietromanuel.ferraro@unicatt.it; 3 Istituto di Medicina Interna e Geriatria, Università Cattolica del Sacro Cuore, 00168 Roma, Italy; 4 Nephrology, Transplant and Dialysis Unit, AOUP, 56126 Pisa, Italy; m.barsotti63@gmail.com; 5 Department of Medicine, University of Verona, 37129 Verona, Italy; giovanni.gambaro@hotmail.it

**Keywords:** kidney stone, nephrolithiasis, diet, prevention, fluid therapy, nutrition

## Abstract

Kidney stone disease should be viewed as a systemic disorder, associated with or predictive of hypertension, insulin resistance, chronic kidney disease and cardiovascular damage. Dietary and lifestyle changes represent an important strategy for the prevention of kidney stone recurrences and cardiovascular damage. A full screening of risk factors for kidney stones and for cardiovascular damage should be recommended in all cases of calcium kidney stone disease, yet it is rarely performed outside of stone specialist clinics. Many patients have a history of kidney stone disease while lacking a satisfactory metabolic profile. Nonetheless, in a real-world clinical practice a rational management of kidney stone patients is still possible. Different scenarios, with different types of dietary approaches based on diagnosis accuracy level can be envisaged. The aim of this review is to give patient-tailored dietary suggestions whatever the level of clinical and biochemistry evaluation. This can help to deliver a useful recommendation, while avoiding excessive dietary restrictions especially when they are not based on a specific diagnosis, and therefore potentially useless or even harmful. We focused our attention on calcium stones and the different scenarios we may find in the daily clinical practice, including the case of patients who reported renal colic episodes and/or passed stones with no information on stone composition, urinary risk factors or metabolic cardiovascular risk factors; or the case of patients with partial and incomplete information; or the case of patients with full information on stone composition, urinary risk factors and metabolic cardiovascular profile.

## 1. Background

Kidney stone disease is quite prevalent in western countries: in the United States, its prevalence in a representative sample of the general population reaches 9% [1] and similar figures have been reported in European populations [2]. It shows also a high recurrence rate, namely 30% to 50% at 5-years [3,4]. Therefore, primary and secondary prevention of urinary tract stone disease is an important medical task.

A history of kidney stones is also associated with a higher prevalence of chronic kidney disease and cardiovascular damage or events when compared with non–stone formers [5,6,7,8]. It has been suggested that insulin resistance may be the link between kidney stones and cardiovascular disease [9]. 

The association between kidney stones and metabolic syndrome and/or cardiovascular disease should prompt physicians to consider the assessment of the cardiovascular risk factors in any adult with kidney stones. The final goal should be an early prevention of cardiovascular, bone and kidney damage [10,11] other than kidney stone recurrences.

Kidney stone disease should be viewed as a systemic disorder [12,13], associated with or predictive of hypertension, insulin resistance, chronic kidney disease, metabolic bone disease and cardiovascular damage [14]. All these conditions negatively impact patient’s prognosis and quality of life [10,12]. Hence, it emerges that kidney stone patients need a systemic approach, not only limited to urinary tract stone treatment or prevention.

Dietary and lifestyle changes are a major strategy for the prevention of kidney stone recurrences. It is noteworthy that several risk factors for kidney stone formation as well as for cardiovascular damage are modifiable and related to lifestyle and dietary habits [15].

Dietary intervention [16] aims to correct urinary abnormalities known to induce lithogenesis, but also to prevent weight gain, hypertension, diabetes or obesity.

Increasing body mass index (BMI) was associated with increasing urine sodium and decreasing pH in men and increasing urine uric acid, sodium, and decreasing urine citrate in women [17]. Similar to obese stone formers, overweight stone formers show alterations in metabolic urinary profiles that are associated with increased overall risk of stone formation [17].

Looking at the existing literature in the field of nutritional management for kidney stone patients, several papers and guidelines propose schematic dietary suggestions related to urine abnormalities while others report the effects of nutrients and food categories on different urinary risk factors [18,19,20]. Therefore, they assume that a full urinary metabolic evaluation is always available. In addition, they rarely underline the need for dietary and lifestyle recommendations to reduce the cardiovascular risk. 

Based on their clinical feature and risk of recurrence, kidney stone patients should undergo basic evaluation (including medical history and physical examination, ultrasound, blood analysis and urinalysis), or a full metabolic assessment (including 24-h urine metabolic evaluation) [19]. Unfortunately, the real clinical practice is quite different from this ideal scenario. For many kidney stone formers, stone composition is often unknown (or sometimes defined as calcium-based or radiolucent) and a full metabolic work-up including 24-h urine assessment is seldom performed outside of selected kidney-stone centers with motivated and trained urologists or nephrologists. For example, a recent retrospective study showed that only about 8% of high-risk stone formers are offered a full metabolic evaluation [21]. Instead, general practitioners and patients themselves do not undergo any metabolic screening after passing a kidney stone. The result is that kidney stone patients rarely receive a diagnosis of the type of stones and of the urinary risk factors profile. Unfortunately, this limits the chances of an effective and safe implementation of preventive dietary intervention, and even may favor stone recurrences and unwanted side effects.

So, based on the observed clinical practice experience, in this paper we try to reproduce what may happen in the clinical practice and suggest a pragmatic dietary approach to kidney stone patients in three possible scenarios (Table 1).

A precise diagnosis of stone composition and of urinary risk factors is the basis for a successful treatment and prevention: this should be done for all the kidney stone patients. Namely, analysis of stone composition preferably by infrared spectroscopy and metabolic evaluation, including blood biochemistry and a 24-h urine collection for urinary excretion of calcium, oxalate, citrate, sodium, magnesium, and creatinine should be the gold standard for a kidney stone former assessment [13,22]. Although recently an empirical approach of kidney-stones patients has been suggested, which does not require any definition of the clinical and biochemical phenotype [23], many believe that the greater the accuracy of the diagnostic assessment, the greater is the specificity, safety and successfulness of the dietary (and pharmacological) interventions [24].

Unfortunately, this rarely occurs in the daily clinical practice. Lack of information and feasibility, costs, poor motivation of physician and patients, are only some of the barriers to the implementation of full metabolic screening for kidney stone disease. Wertheim et al. [25] reported that the majority of urologists said they should provide dietary recommendations regardless the rate of recurrence of stone events. Unfortunately the small amount of time dedicated to nutrition, 4–10 min per patients, is likely inadequate [25]. 

When an accurate diagnosis, including assessment of 24-h urine solute excretions, is not available or possible, physician and dietician must limit the dietary advice to non-specific suggestions, i.e., increase fluid intake and have a healthy diet while avoiding all misconceptions existing amongst the general population, health professionals and physicians about dietary management of stones. For example, reducing calcium in the diet without a diagnosis of diet-dependent hypercalciuria can induce osteopenia and increase oxalate absorption leading to a high (rather than reduced) risk of calcium-oxalate stone formation. Similarly, reduction of dietary oxalate without a diagnosis of enteric hyperoxaluria may reduce the intake of many vegetables leading to lower alkaline load and fiber intake; in turn this has negative effects on stone formation (namely reduction of citrate and of urinary pH and an increase in calcium excretion) and on many cardiovascular (CV) risk factors (increasing of blood pressure and serum lipids). 

Consensus exists that the first line for the non-specific prevention of kidney stones is a high fluid (water) intake, provided the exclusion of urinary tract obstruction. 

## 2. Fluid Therapy

Low hydration is a risk factor for all type of stones, whereas ensuring a urine volume of at least two liters per day reduces the risk of urine oversaturation. To obtain this goal, fluid intake may vary in function of the extrarenal loss of water as that of the sweating, breathing and perspiratio insensibilis etc., so the daily fluid intake may vary from 1.5 to 3 L. 

The Guideline of the American College of Physicians [26] as regard the effect of increased branded water intake versus increased tap water intake on stone recurrence cited only one study that tested the effect of a particular brand of water containing 15 mg/L of calcium versus tap water with 55 to 130 mg/L of calcium; 17% of stone formers who drank 2 L of oligo-mineral water had recurrences versus 22.9% of those who drank the same volume of tap water [27]. No other study investigated the effect of branded oligo-mineral waters on stone recurrence rates. 

Water mineral composition may represent a modifiable factor for preventing stone formation but an adequate volume is the main goal. The exact daily amount of fluids needed by stone formers is not clearly defined. The most proper suggestion for our patients should not be how much fluids to introduce but how much the urine volume should be, and educate them to measure periodically (for example at the change of season). The prescription of drinking in order to form at least 30 mL/kg of body weight of urine per day may be recommended [28], which may be higher than 2 L per day. 

It can happen that people ask: “*I cannot drink all this water! May I drink something else? The important thing is that it is a fluid, correct*?” A recent study investigated the effect of different beverages on the risk of kidney stones. Higher consumption of sugar-sweetened soda, in particular those containing fructose, was associated with a higher incidence of kidney stones [29]. Fructose has been shown to favor uric acid synthesis [30] and to increase the urinary excretion of calcium, oxalate and uric acid [31,32]. A high fructose intake can promote the production of uric acid. Cell fructose uptake uses ATP very rapidly. Fructose induces nucleotide turnover: an excessive intake of this monosaccharide decreases intracellular phosphate and stimulate uric acid generation. Fruit is rich in fructose so in the case of hyperuricuria it is appropriate to suggest avoiding an excess of fruit intake (no more than 2–3 serving per day). Special attention must be paid to processed foods with fructose such as beverages, home-made fruit juice and baby food and to the use of fructose as sweetener.

Shuster et al. [33] showed that cola drinks consumption was associated with stone formation and recurrence possibly because of acid-phosphoric contents that is typically added to cola drinks. On the contrary, acid-citric soda could have the potential to reduce the stone risk by increasing urinary citrate excretion [34]. However, in large prospective cohort studies, consumption of low-energy carbonated beverages was not significantly associated with stones, suggesting a role for fructose rather than carbonation per se as the culprit for stone formation [29].

Moreover, mono and disaccharides intake with these beverages also has a negative impact on the glyco-metabolic profile and weight control, thus increasing the risk of metabolic disorders that in turn may predispose one to stone formation. 

Coffee and tea were associated with a decreased risk of stones in the Channing cohorts [29] possibly due to increased urine volume output for the caffeine content even if a similar effect had been shown also with decaffeinated coffee and tea. In any case, excessive consumption of coffee and tea should not be encouraged in order to not interfere with blood pressure control. 

Alcohol, and beer in particular, seems to have a protective effect probably because it inhibits antidiuretic hormone secretion, decreasing urinary salt oversaturation [29]. Drinking grapefruit and cranberry juices should be discouraged because they are rich in oxalate [29]. Similarly beer should be avoided in the case of hyperuricosuria because of its purine content [35].

“*When and how to drink*?” The intake should be distributed appropriately throughout the day, in particular in the most critical periods as after a meal, during the night, in the event of sweating (physical activity, climate too hot) or gastrointestinal losses (diarrhea or vomiting) [36].

“*Which kind of water*?” Rodgers found that mineral water containing calcium and magnesium should be considered as a possible therapeutic or preventive measure in calcium oxalate kidney stone disease [37]. 

Not all fluids have beneficial effect in reducing the risk of kidney stones [29]. For instance, lemonade and fresh lemon juice seem to increase significantly citrate excretion, reduce urinary calcium excretion with no change in oxaluria. Orange and grapefruit juices increase citraturia more than lemonade and increase oxaluria but this was not associated with a greater oversaturation of calcium-oxalate. Lime juice increase urine citrate, potassium and pH. With regard to non citrus juice, the consumption of cranberry juice is controversial as regard modification in urinary citrate excretion but it increases calcium and oxalate excretion [38]. Also, blackcurrant and melon juices have positive effects in terms of increasing citraturia. Soda consumption seems not to influence citraturia while the effect of citrus-based sports drink changes depending on the brand [20,39].

A limitation is that citrate content in juice and beverages is not available from nutrients composition tables. In any case, as already mentioned, attention should be paid to the consumption of high amount of citrus or non-citrus and sweetened beverages because of the simple sugar fructose content and the extra-energy supply that may predispose to unfavorable outcomes [10,40]. 

## 3. Healthy Diet

In the absence of a known CV and/or kidney stone risk profile guidelines for proper nutrition should be suggested. The key aspects are 1. reducing simple sugars intake while preferring complex carbohydrates; 2. reducing saturated fats while preferring vegetable, mono and poly-unsaturated fats; 3. reducing the use of salted and packaged and preserved foods preferring the fresh ones; 4. increasing the consumption of fruit, vegetables and food rich in fiber; 5. reducing animal protein intake while preferring vegetable sources; 6. maintaining or gaining an ideal body weight and having an active lifestyle.

DASH (DASH, Dietary Approaches to Stop Hypertension) and Mediterranean diets represent healthy dietary patterns applicable to the general population: they have an additional favorable role in the prevention of kidney stones formation as reviewed by Borghi et al. [41]. Taylor et al. [42] examined the relation between DASH diet and the risk of incident kidney stones and showed a significant decrease in the risk of incident kidney stone formation regardless of sex, age and BMI in those following the DASH diet [42]. 

(1) Reduce simple sugars intake while preferring complex carbohydrates: An acute load of glucose causes an increase of calcium urinary excretion in normal subjects which is greater in calcium stone formers. Furthermore, diets with different content of carbohydrates at equal supply of other nutrients causes an increase in calcium excretion directly proportional to carbohydrates intake [43]. A good glyco-metabolic control in type II diabetes has been shown to significantly decrease calcium excretion. As reported above evidence exists of a strict relationship between insulin resistance and nephrolithiasis, and uric acid stones disease in particular [44].

The association between insulin resistance and uric acid stones disease can be explained by a defect in urine acidification, namely to an impaired L-glutamine system and reduction of Na+-K+ transport at the proximal tubule [44]. This change results in very low urine pH leading to critical urine oversaturation for uric acid. Insulin resistance is associated with a defect of the Na+/K+ and H+ transport systems [45] that can cause reduction of citrate excretion [46] and, on the other hand, insulin may also affect renal handling of calcium and phosphate [47]: as a whole, it is well in keeping with increased risk of calcium stone formation. So, it is not surprising that insulin resistance favors formation of uric acid and calcium stones and that they are likely associated with metabolic syndrome, overweight condition, arterial hypertension, diabetes [48]. 

(2) Reduce saturated fats while preferring vegetable, mono and poly-unsaturated fats. Stone formers have a high prevalence of hypercholesterolemia and the amount of fat introduced is directly proportional to oxalate excretion. Arachinodic acid in particular seems to be implicated in stone formation. Arachidonic acid is the precursor of prostaglandins. Prostaglandin E_2_ (PGE_2_), above all, causes hypercalciuria in a number of ways: it increases intestinal calcium absorption; it decreases its reabsorption in the renal tubules; and it increases bone resorption [49]. 

Yasui et al., observed a significant decrease in urinary calcium and oxalate and an increase of urinary citrate after the administration of 1200 mg of fish oil per day [50].

However, the effect of omega 3 intake and supplementation on the lithogenic risk for recurrent nephrolithiasis seem all in all limited [51].

(3) Reduce the amount of salt and preserved foods. High salt intake is associated with an increase of urine calcium excretion. It has been estimated that an increase of 100 mmol of sodium chloride (6 grams of salt) increases urinary calcium of 1mmol in normal subjects and a two-fold increase in hypercalciuric stone formers. These results come both from salt-loading studies and intake studies on the general population. Nouvenne et al. carried out a randomized controlled trial on calcium stone formers for a period of three months and found that a low-salt dietary regimen corrected idiopathic hypercalciuria in 30% of the cases and seemed to have a positive effect also on other urinary stone risk factors [52]. Moreover, high levels of dietary sodium are associated with raised blood pressure and adverse cardiovascular health.

There is a worldwide effort to carry out policies for salt intake reduction in the general population. However, all previous observations suggested a reduction in excessive salt consumption in stone formers too. In Europe and North America over 75% of salt comes from processed foods or foods prepared in restaurants, 10–12% is naturally present in food and the remaining is added in the kitchen or at the table. The amount of salt recommended by the dietary guidelines is 5–6 g per day. This goal can be achieved with a few simple steps such as to reduce or avoid adding salt at the table, to get used to a taste different from that of salt by using spices, avoiding the consumption of foods containing salt as a preservative, pre-cooked meals and packaged foods.

(4) Increase the consumption of fruit, vegetables and food rich in fiber.

A higher fruit and vegetable intake is associated with lower risk of urinary stones [42]. Similar data have also been observed in the EPIC study where high intakes of fresh fruit, fiber from wholegrain cereals and magnesium were associated with a lower risk of kidney stones compared with a high meat diet [53]. It was also observed that vegetarians have half the risk of omnivores to form stones [54]. 

Sorensen et al. [55] too found that high dietary intake of fiber, fruits and vegetables were associated with a reduced risk of incident kidney stones in postmenopausal women but not in those women with a history of stones. Notwithstanding this possible caveat, the body of data suggests a higher intake of fruit and vegetables in stone formers as those who eat more fruit and vegetables are often subjects who make good dietary choices and have a healthy lifestyle.

The mechanism of the protective effect on the stone risk of a diet rich in fruits, vegetables and fibers is multifaceted. A higher intake of fruit and vegetables may result in a higher urinary oxalate excretion and a greater risk for calcium oxalate nephrolithiasis because of their high oxalate content. However, consumption of fruits and vegetables even more strongly increases urinary citrate, an important inhibitor of lithogenesis thus counteracting the dismal effect on oxaluria. 

Fruits and vegetables, are also an important source of water, magnesium and potassium, and represent an alkali load that further increases the urinary excretion of citrate [56]. The alkali content in fruit and vegetables may offset the acid load brought by animal protein, resulting in a less acidic urine milieu and thus in a reduced propensity to lithogenesis [57]. 

Fruit and vegetables together with other food groups rich in fibers such as cereals and legumes have a high content of phytate which is thought to have a protective effect against stone formation. Curhan et al. found an association between higher dietary intake of phytate and lower risk of stones in a cohort of women taking part in the Nurses Health Study II [58]. 

In the gastrointestinal tract phytate forms insoluble complex with calcium favoring oxalate intestinal absorption [59]. Despite this, phytate seems to play a role in the prevention of calcium stones formation inhibiting urinary crystallization of calcium salts; actually it seems to be absorbed and excreted in the urine [60,61,62].

(5) Reduce animal protein intake while preferring plant-derived proteins 

Animal proteins are rich in purines, sulfur amino acids, oxalate precursors. Moreover, food containing animal proteins are also rich in phosphorus. A high animal protein intake causes renal hyperfiltration, increases urinary uric acid, calcium, oxalate, phosphate excretion, reduces urinary pH and citrate excretion: as a consequence, the overall risk of kidney stones formation rises [28]. Uricosuria and phosphaturia increase as a direct result of high intake of purines and phosphorus. Low citraturia and pH are secondary to the acidifying effect of sulfur aminoacids, while the increase in oxaluria is probably due to an increased endogenous production as animal proteins are rich in precursors of oxalate (tryptophan, phenylalanine, tyrosine and hydroxiprolina) [62,63]. Hypercalciuria secondary to high animal protein intake results from higher bone reabsorption and a lower tubular calcium reabsorption [64]. Reddy et al. showed that a low-carbohydrate high-protein diet for 6 weeks produces a high renal acid load, increases the risk for stone formation, and decreases calcium balance thus increasing the risk for bone mass loss [65]. However, the negative effect of high protein diets on bone status was not corroborated by the Cao et al. study [66]. These authors showed that a similar diet also increases calcium absorption, partially compensating the increase in urinary excretion without changing bone resorption. Most likely the role of protein on calcium balance and bone turn-over is more complex and dependent on other dietary factors (i.e., alkalizing factors) [43,67]. 

A diet based on vegetable proteins have different effects on lithogenesis. In normal subjects a flesh based diet increases the risk for uric acid stones as compared to a vegetable-based protein diet with the same protein and electrolytes content [64]. This is probably due to lesser urinary calcium, uric acid, and higher oxaluria and citraturia because of the lower content of purines, sulfur amino acids, phosphate in vegetables [41].

(6) Maintain or gain an ideal body weight and have an active lifestyle.

Both obesity and high energy intake increase the risk of stone formation and incident stones [55]. Inconsistent results of the effect of physical activity have been observed. While Sorensen et al., disclosed a reduction of the risk of incident kidney stones in postmenopausal women independently from energy intake and BMI [55], Ferraro et al. in the Channing cohorts did not observe any reduction associated with exercise [68].

**Figure 1:**
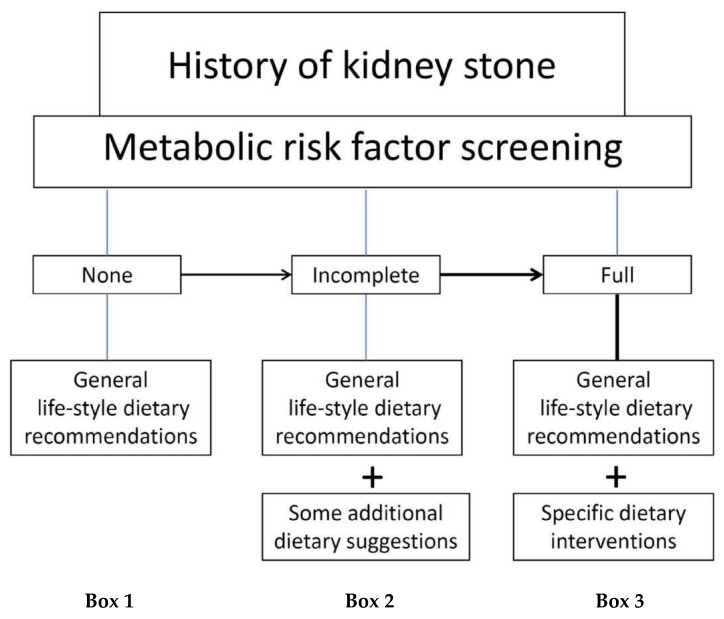
Different type of dietary approach (see Box 1–2–3) on the basis of the diagnosis accuracy level.

It is noteworthy that kidney stone disease is associated with CV morbidities [69]. Whatever the effect on lithogenesis, regular physical activity should be systematically suggested to all renal stone patients. Following are the most suitable dietary suggestions in the three scenarios outlined in Figure 1 on the basis of the previous considerations on the relationship between diet and stones formation. 

(a) Patients who reported renal colic episodes and/or passed stones with no information on stone composition and/or on urinary risk factors and/or on metabolic cardiovascular risk factors.

This is the case when no information about the stone composition and the urinary risk factors is available. In this case, one can only promote a healthy diet and high fluid intake, and regular physical activity (Box 1).

Box 1  

*Practical suggestions*

- Healthy diet
- Physical activity
- Fluid therapy

Briefly, reduction of the intake of simple sugars and animal fats while preferring complex carbohydrates and vegetable fats, limitation of salt intake and conserved foods, preferring vegetables-derived proteins instead of animal proteins, increasing the intake of fruits, vegetables and foods rich in fibers. These tips are the main rules for a healthy diet and have been shown to exert a favorable effect on the lithogenic and CV risk. Physical activity is part of a healthy lifestyle; it helps to maintain or gain an ideal body weight. Good hydration is another mandatory recommendation. It is important to explain to the patient that the goal of good hydration is a urinary volume of 2 or more liters per day to avoid super-saturation. Even if specific information is not available a patient-centered approach is preferable; this means to start from patient’s habits and needs and suggest gradual changes to favor adherence to prescriptions. 

A Decalogue summarizes the proposed non-specific pragmatic approach to kidney stone patients (Table 2). 

(b) Patients who formed/passed stones with only incomplete information on stone composition (calcium or not calcium stones) or on urinary risk factors or on metabolic cardiovascular risk factors.

This is the case when we have only partial information, more often including the presence or not of calcium at plain Rx film, or the measurement of 24-h urine calcium or uric acid. Recommendations described in point (a) at the aim of changing patient lifestyle in order to ameliorate dietary habits, increase regular physical activity, achieve better weight control and reduce cardiovascular risk factors should be applied. On top of this, further specific instructions can be added according to available data, generally 24-h urinary calcium and/or uric acid data (Box 2).

Box 2  

Practical suggestions

- Healthy diet associated to physical activity
- Fluid therapy

Plus, in the case of Hypercalciuria

- low salt (5–6 g/day) and normal protein (0.8 g/kg body weight) intake, with the preference of proteins from plant origin, namely legumes and cereals. These suggestions are included in the healthy diet recommendations
- Ensure an adequate calcium intake (1000–1200 mg/day)

Hyperuricosuria

- Reduce foods with high purine content (Table 3)
- Limit fats intake (this suggestion is included in the healthy diet)
- Limit fructose intake, especially processed foods (sweetened beverages, baby foods)
- Avoid alcohol intake
- Normal protein intake, with the preference of protein from plant origin, namely legumes and cereals

If hypercalciuria is observed, dietary indications include reduction of salt intake and normalization of protein intake. In hypercalciuric stone formers with a normo-calcic diet (about 1g/day), decreasing salt intake reduced calcium excretion [70]. Thus, a restriction of dietary salt intake (i.e., a normal salt intake as recommended in the healthy diet, that is 5–6 g/day) could be suggested for primary and secondary prevention of calcium stone formation. An excessive intake of animal protein enhances urinary calcium excretion. A normo-protein diet (0.8 g/kg BW) together with a normal calcium intake (800–1000 mg/24-h) decreased urinary calcium together with many bone and stone risk factors in hypercalciuric stone formers and in non-stone formers [43,71]. 

If hyperuricosuria is detected, reduction in purine-rich foods and in animal protein intake lowers uric acid excretion. Greater effects were found if the reduction in purine intake was associated with a healthy diet [72]. Foods rich in purine are shown in Table 2 [73].

**Table 3:** Foods grouped in three categories by purines content per 100 g of edible part.

High	Moderate	Low
>500 mg/100 g	400–100 mg/100 g	<100 mg/100 g
Liver	Asparagus	Coffee
Kidney	Chicken	Bread
Thyme	Crabs	Pasta
Anchovies	Duck	Rice
Sardines	Ham	Eggs
Herring	Beans	Milk and dairy
Mussels	Lentils	Sugar
Smoked bacon	Mushrooms	Tomato
Trout	Lobster	Green vegetables
Cod	Oyster	
Lamb	Pork	
Goat	Shrimp	
Game	Spinach	

The suggestion of a healthy diet is also useful because it entails the reduction of animal fat intake and potentially decreases ketogenesis. The hydroxybutyric and acetoacetic acids in particular compete with uric acid at tubular level, and being stronger acids than uric acid, are more easily eliminated by the kidney causing an increase of uric acid serum levels but a lowering urine pH which can favor the precipitation of the uric acid in the urine [74].

A high fructose intake promotes uric acid synthesis. Cell fructose uptake uses ATP very rapidly. Fructose induces nucleotide turnover: an excessive intake of this monosaccharide decreases intracellular phosphate and stimulates uric acid generation. Since fruit is rich in fructose, in case of hyperuricosuria it is proper to suggest avoiding an excess of fruit intake (no more than 2–3 servings per day). Special attention must be paid to processed foods with fructose such as beverages, home-made fruit juice and baby food and to the use of fructose as sweetener. Industrial fruit juice generally has a lower content of fruit (<60%) and fructose (obviously if it is not added as sweetener).

Reducing alcohol consumption may also have positive effects. The ethyl alcohol is oxidized to acetaldehyde in the liver with reduction of NAD^+^ to NADH subtracting it from glyceradeide-3-phosphate dehydrogenase, thus slowing down glycolysis. The alternative way for energy production is fatty acids oxidation which, in these conditions, leads to ketone bodies production with the previously described consequences.

To avoid uric acid crystal formation in urine it is important to obtain an alkalizing environment. This can be obtained with the dietary tips previously described: reduction of animal protein preferring protein from plant origin (namely legumes plus cereals); increase in vegetables food (but not overdoing with fruits to limit fructose intake). 

(c) Patients who formed/ passed calcium stones and with full information about stone composition, urinary risk factors and metabolic cardiovascular profile.

In the case when a full metabolic assessment is available, we can give targeted information to the patient. It is important to remember that the following instructions must be considered in addition to a healthy diet and a proper fluid therapy (Box 3).

Box 3  

*Practical suggestions*

- Healthy diet associated to physical activity
- Fluid therapy

Plus, in the case of Hypercalciuria

- If hypercalciuria is diet-dependent a low calcium intake (400 mg/day) is recommended
- If hypercalciuria is not diet dependent, a calcium intake restriction is not recommended and an adequate calcium intake (1000–1200 mg/day) should be ensured
- low salt (5–6 g/day) and normal protein (0.8 g/kg body weight) intake, with the preference for proteins of plant origin, namely legumes plus cereals. These suggestions are included in the healthy diet recommendations

Hyperoxaluria

- Avoid foods with a high content of oxalate (Table 3)
- Do not avoid fruit and vegetables
- Ensure an adequate calcium intake (1000–1200 mg/day)
- Avoid excessive intake (>500 g/day) of ascorbic acid (Vitamin C), vitamin C supplements in particular

Hypocitraturia

- In addition to proper fluids therapy and healthy diet
- Increased fruit and vegetables
- Moderate intake of citrus based beverage paying attention to simple sugar and energy intake

## 4. Hypercalciuria

In this case, dietary calcium restriction is only appropriate in the case of diet-dependent hypercalciuria. A low calcium diet (440 mg/24-h) in calcium stone formers decreased calciuria but increased the urine oversaturation of calcium-oxalate in hypercalciuric patients [75]. In hypercalciuric stone formers a diet with a calcium content ≥1g/24-h reduces the risk of stone formation, while low calcium diet reduces urinary calcium but significantly increases the oversaturation of calcium-oxalate [20].

Other recommendations to reduce hypercalciuria have already been described. 

## 5. Hyperoxaluria

Oxalate is the final product of glyoxylate oxidation, an intermediate of glycine metabolism and is abundantly present in vegetables. In normal conditions the amount of oxalate from endogenous metabolism and from the diet is low and daily oxaluria ranges from 15 to 30 mg. Approximately 50% comes from the diet. Its solubility depends on urinary pH and the presence of calcium ions and it is generally completely dissociated in the urine. Increased excretion of urinary calcium can cause calcium-oxalate crystals formation. The intestinal absorption of oxalate depends on the availability of free calcium in the intestinal lumen. If this is reduced (low calcium diet, malabsorption, etc.) oxalate can be easily absorbed [76]. On the other hand, reducing oxalate in the diet increases intestinal calcium absorption. Therefore, controlling oxalate-rich foods (Table 4) [77] intake may be advisable in case of hyperoxaluria, but limiting fruit and vegetables consumption is not wise. The reason is not simply because of the general positive effects of a healthy diet, but also for the specific effect on the urine stone risk that a dietary restriction of fruit and vegetables would have. In fact, it can induce a moderate increase of calcium absorption and excretion, and cause a decrease of crystallization protective molecules such as magnesium, citrate, potassium [70]. Meanwhile, the addition of fruit and vegetables in stone formers with low levels of urinary citrate didn’t change oxalate excretion while it did reduce the lithogenic risk because of the increase in urinary magnesium, citrate, potassium, pH and volume [78].

An important suggestion to give to stone patients is that oxalate rich food assumption should go together with calcium containing foods. In particular, they should be warned that some energetic nutritional supplements taken during or after physical performances are very rich in oxalate (almonds, grain, chocolate as such or in the form of bars) and poor in calcium. These power bars can induce the over-excretion of oxalate when urine volume is low because of the exercise induced sweating, thus favoring calcium-oxalate crystallization.

Ascorbic acid degradation results in oxalate formation. The breakdown of 60 mg of ascorbic acid to oxalate potentially results in the formation of up to 30 mg oxalate per day. Taylor et al. [76] has found that with even low levels of dietary ascorbic acid consumption, small increases in intake (>281 mg/day vs. <105 mg/day) increased stone risk by 31% in a cohort of male health professionals. 

Taylor et al. [76] and Thomas et al. [79] reported that total ascorbic acid intake increased stone risk two fold in a large Swedish population. Individuals constantly consuming large oral doses of ascorbic acid have been reported to develop oxalate nephropathy in several case reports [80]. 

The hypothesis was advanced that it is possible to modulate gut oxalate degradation by the intestinal microbiota. However, in a study using supplementations of the probiotic lactobacillus no reduction in oxalate urinary excretion was observed [81]. Further studies are needed to clarify the effects of probiotics, other than lactobacillus, in calcium-oxalate stone formers with hyperoxaluria.

## 6. Hypocitraturia

Hypocitraturia is a common metabolic alteration in kidney-stone formers. Renal excretion of citrate is strictly linked to acid-base status. The acid-base balance is regulated by the net endogenous acid production in relation to acidifying and alkalizing foods and by organic acids metabolism. Fruits and vegetables are the main source of dietary alkalinizing compounds while foods rich in animal proteins such as meat, poultry, fish, eggs, have an acidifying effects due to the presence of sulfuric amino-acids. Hypocitraturic patients generally have a low intake of vegetables. A high intake of fruit and vegetables (excluding those rich in oxalate), increased citrate excretion and reduced urinary saturation for calcium oxalate and calcium phosphate [20,78]. As regards the effect of beverages on urinary citrate excretion see the paragraph on fluids therapy.

## 7. Practical Applications 

A full screening of risk factors for kidney stones and for cardiovascular damage should be recommended in all cases of calcium kidney stone disease, but it is quite rarely performed outside of specialized stone clinics. Many patients who have a history of kidney stone disease do not undergo a complete metabolic profiling. However, in this real-world clinical practice, it is still possible to rationally manage kidney stone patients. Different scenarios, with different types of dietary approaches, on the basis of the diagnosis accuracy level can be envisaged (Figure 1). 

In this review we gave dietary suggestions tailored to individual patients whatever his/her level of clinical and biochemistry evaluation. This approach can help to deliver useful recommendations, avoiding excessive dietary restrictions that may not be based on a diagnostic assessment, and which therefore may be potentially useless or even harmful. 

## Figures and Tables

**Table 1:** Three possible scenarios of clinic presentation of patients with history of kidney stone patients.

**(A) Patients who reported renal colic episodes and/or passed stones, but with no information on stone composition or on urinary risk factors or on metabolic cardiovascular risk factors**
**(B) Patients who formed/passed stones with only partial information on stone composition (calcium or not calcium stones) or on urinary risk factors or on metabolic cardiovascular risk factors**
**(C) Patients who formed/ passed calcium stones and with full information about stone composition and/or urinary risk factors and metabolic cardiovascular profile**

**Table 2:** A Decalogue for general healthy counseling in calcium stone patients.

1	Maintain urinary volume over 2 L/day
2	Limit salt intake to 6 g/day
3	Limit animal protein intake
4	Prefer proteins from vegetable sources
5	Do not avoid milk, yogurt, fresh cheeses
6	Consume plant foods avoiding foods with high oxalate content
7	Reduce/do not increase fat body mass
8	Limit the intake of simple sugars, cholesterol and saturated fats
9	Prefer complex carbohydrates and olive oil
10	Promote regular physical activity

**Table 4:** Foods grouped in 4 categories by oxalate content.

Very High >100 mg per Serving	High 26–99 mg per Serving	Moderate 10–25 mg per Serving	Low 5–9 mg per Serving
Whole grain products	Hazelnuts	Beans	Artichokes
Almond	Cashew	Blueberries	Asparagus (cooked)
Cocoa powder	Peanuts	Potatoes	Lettuce
Buckwheat	Milk chocolate	Tomato sauce	Peas
Beet	Carrots	Blackberries	Apples
Rhubarb	Cauliflower	Walnuts	Pear
Spinach	Celery	Prunes (dried)	Melon
Swiss chard	Orange		

Note: We prefer to define these 4 categories instead of giving the amount of oxalate in each food because it can vary greatly between plants of the same species due to differences in the environment where plants have grown (soil composition, climate, etc.) and differences in analytical methods for oxalate determination.
